# Supplementary material for: Metabolites with Anti-Inflammatory Activities Isolated from the Mangrove Endophytic Fungus Dothiorella sp. ZJQQYZ-1
Source: Microorganisms. 2025 Apr 12;13(4):890. doi: 10.3390/microorganisms13040890 (PMC12029632; doi:10.3390/microorganisms13040890)
Supplement: Supplementary file 1 [file microorganisms-13-00890-s001.zip › microorganisms-3576785-supplementary.pdf]

Supporting Information

Metabolites with Anti-inflammatory Activities Isolated from the Mangrove Endophytic Fungus *Dothiorella* sp. ZJQQYZ-1

Zhaokun Li, Junhao Zhu, Ruxue Mu, Chenxi Wang, Yuru Sun, Bingbing Qian, Ning Li* and Yan Chen*

School of Pharmacy, Anhui Medical University, Hefei, 230032, China

*Corresponding authors

E-mail addresses: cychemistry@163.com (Y. Chen); 1993500019@ahmu.edu.cn (N. Li)

Content

Figure S1. ^{1}H NMR spectrum of compound **1** (500 MHz, DMSO-d_6).

Figure S2. ^{13}C NMR spectrum of compound **1** (125 MHz, DMSO-d_6).

Figure S3. HSQC spectrum of compound **1**.

Figure S4. HMBC spectrum of compound **1.**

Figure S5. ^{1}H-^{1}H COSY spectrum of compound **1**.

Figure S6. HRESIMS spectrum of compound **1**.

Figure S7. ^{1}H NMR spectrum of compound **2** (500 MHz, CD_3OD and $CDCl_3$).

Figure S8. ^{13}C NMR spectrum of compound **2** (125 MHz, CD_3OD and $CDCl_3$).

Figure S9. HSQC spectrum of compound **2**

Figure S10. HMBC spectrum of compound **2**.

Figure S11. HRESIMS spectrum of compound **2**.

Figure S12. ^{1}H NMR spectrum of compound **3** (500 MHz, $CDCl_3$).

Figure S13. ^{13}C NMR spectrum of compound **3** (125 MHz, $CDCl_3$).

Figure S14. HSQC spectrum of compound **3.**

Figure S15. HMBC spectrum of compound **3**.

Figure S16. ^{1}H-^{1}H COSY spectrum of compound **3**.

Figure S17. HRESIMS spectrum of compound **3**.

Figure S18. ^{1}H NMR spectrum of compound **4** (500 MHz, $CDCl_3$).

Figure S19. ^{13}C NMR spectrum of compound **4** (125 MHz, $CDCl_3$).

Figure S20. HSQC spectrum of compound **4.**

Figure S21. HMBC spectrum of compound **4**.

Figure S22. ^{1}H-^{1}H COSY spectrum of compound **4**.

Figure S23. HRESIMS spectrum of compound **4**.

Figure S24. ^{1}H NMR spectrum of compound **5** (500 MHz, CD_3OD).

Figure S25. ^{13}C NMR spectrum of compound **5** (125 MHz, CD_3OD).

Figure S26. HSQC spectrum of compound **5.**

Figure S27. HMBC spectrum of compound **5**.

Figure S28. ^{1}H-^{1}H COSY spectrum of compound **5**.

Figure S29. NOESY spectrum of compound **5**

Figure S30. HRESIMS spectrum of compound **5**.

Figure S31. The IC_{50} value of compounds **5** and **7** against LPS-induced NO production in RAW264.7 cells were calculated and presented.

Table S1. The DP4+ evaluation of **3.**

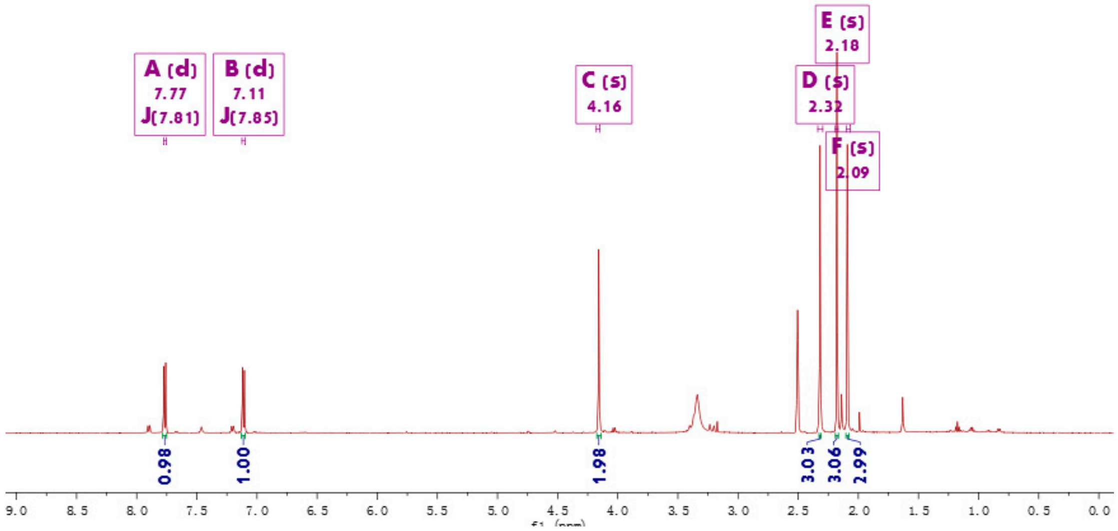

Figure. S1 ^{1}H-NMR spectrum of compound **1** in DMSO-d_6

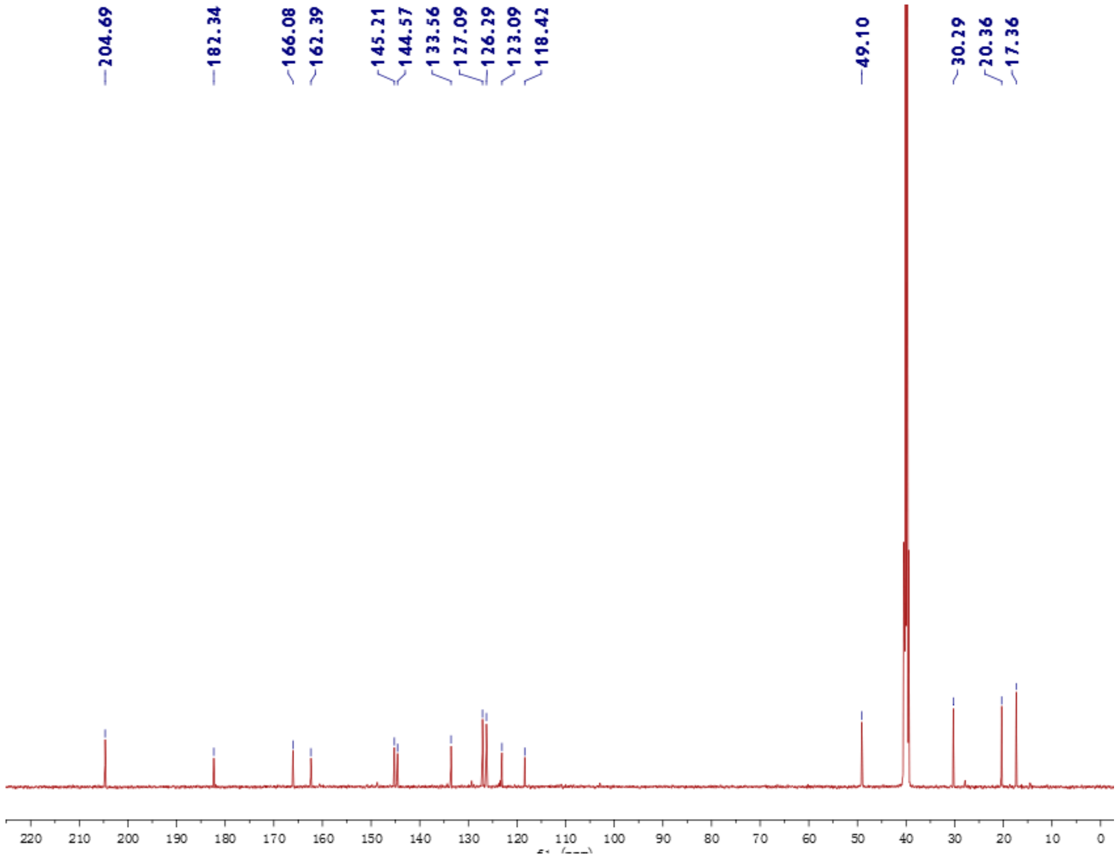

Figure. S2 ^{13}C-NMR spectrum of compound **1** in DMSO-d_6

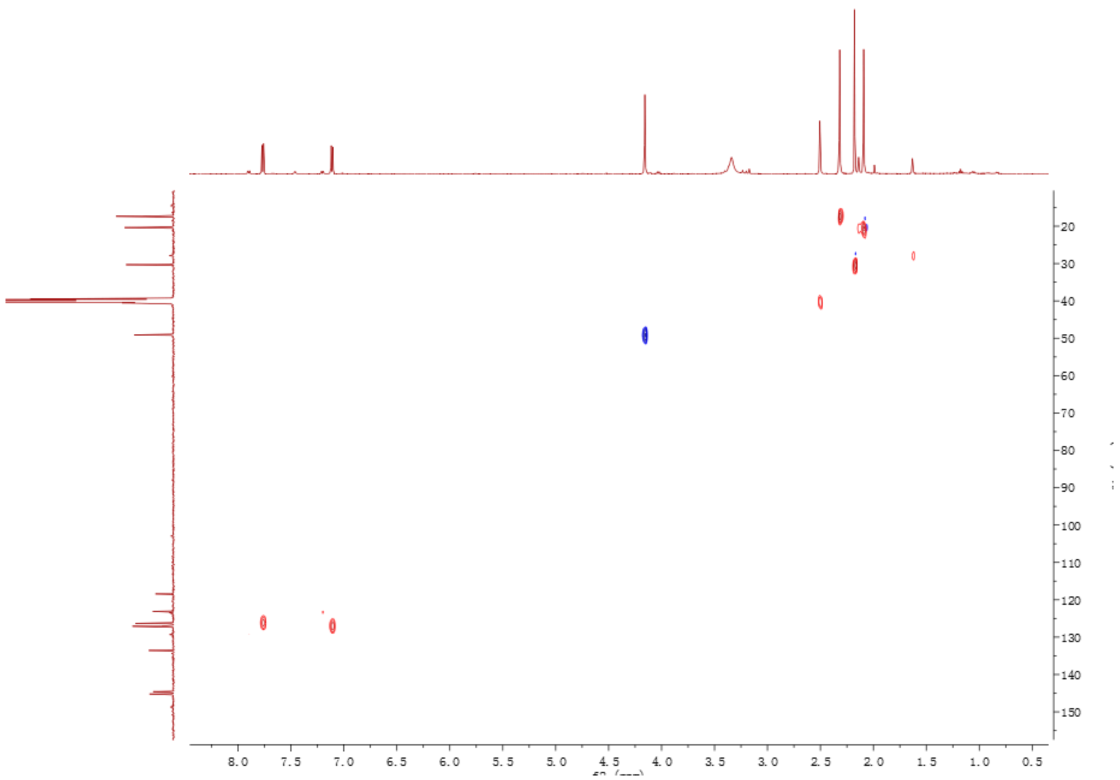

Figure. S3 HSQC spectrum of compound **1**

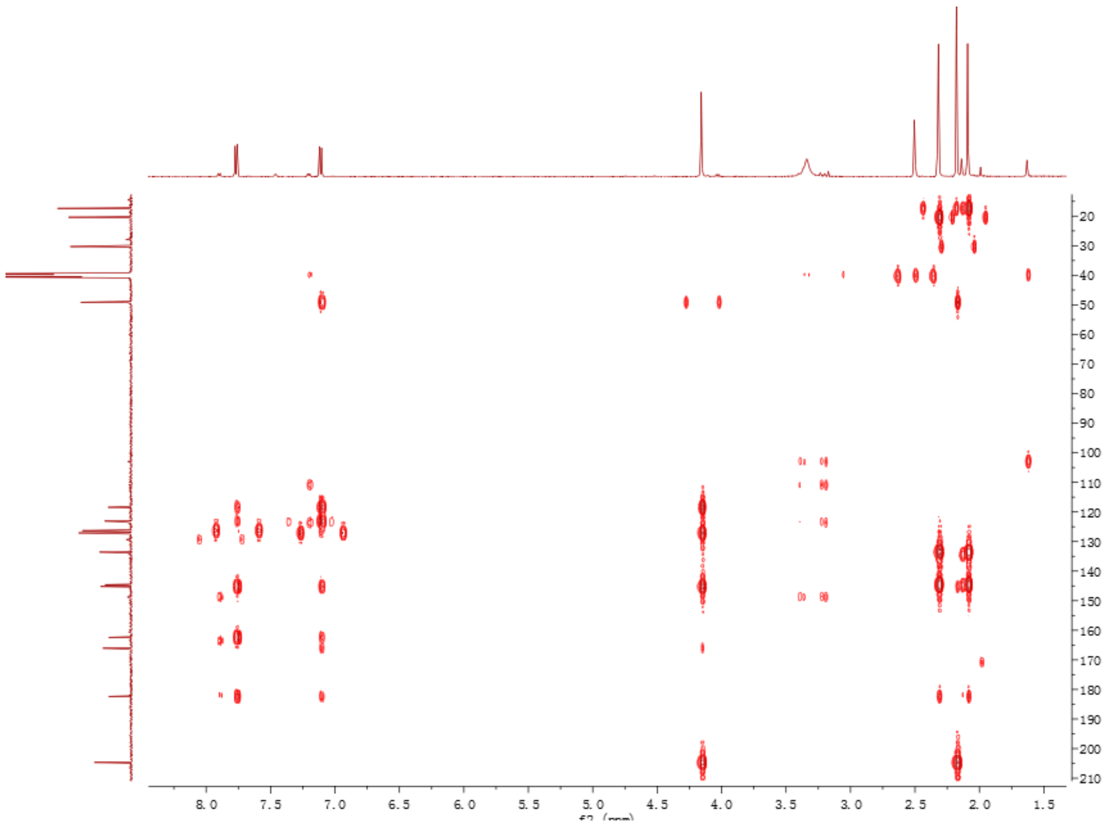

Figure. S4 HMBC spectrum of compound **1**

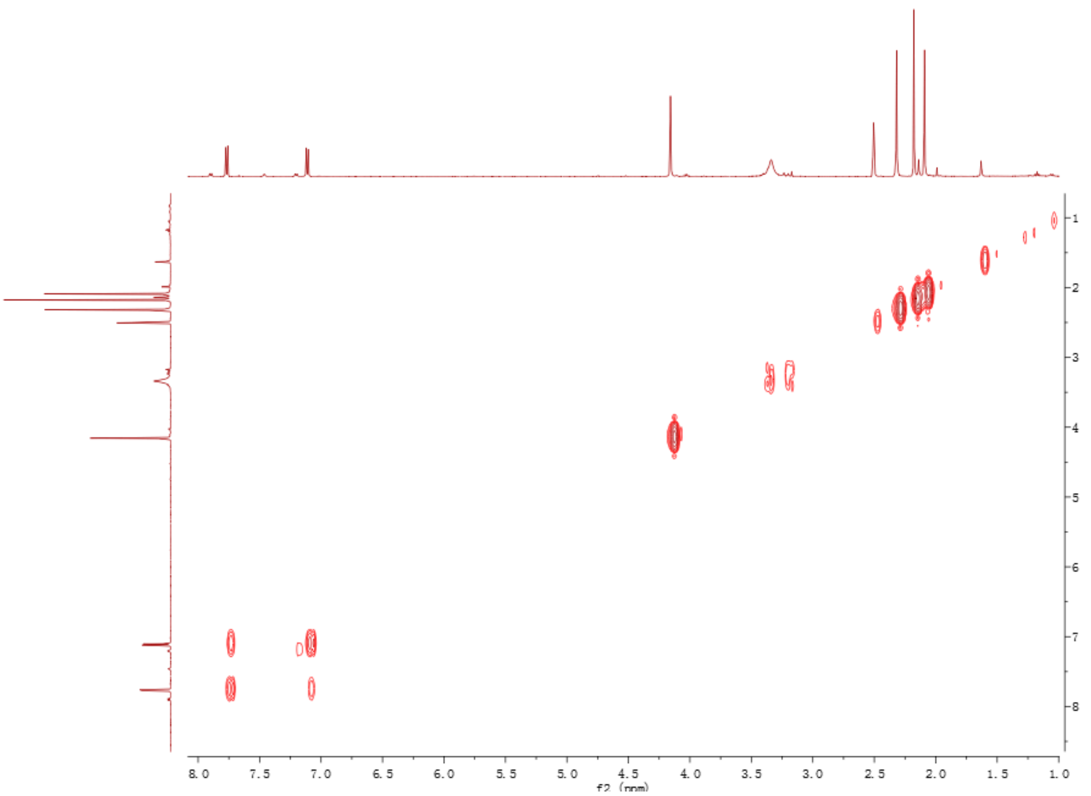

Figure S5 ^{1}H-^{1}H COSY spectrum of compound **1**

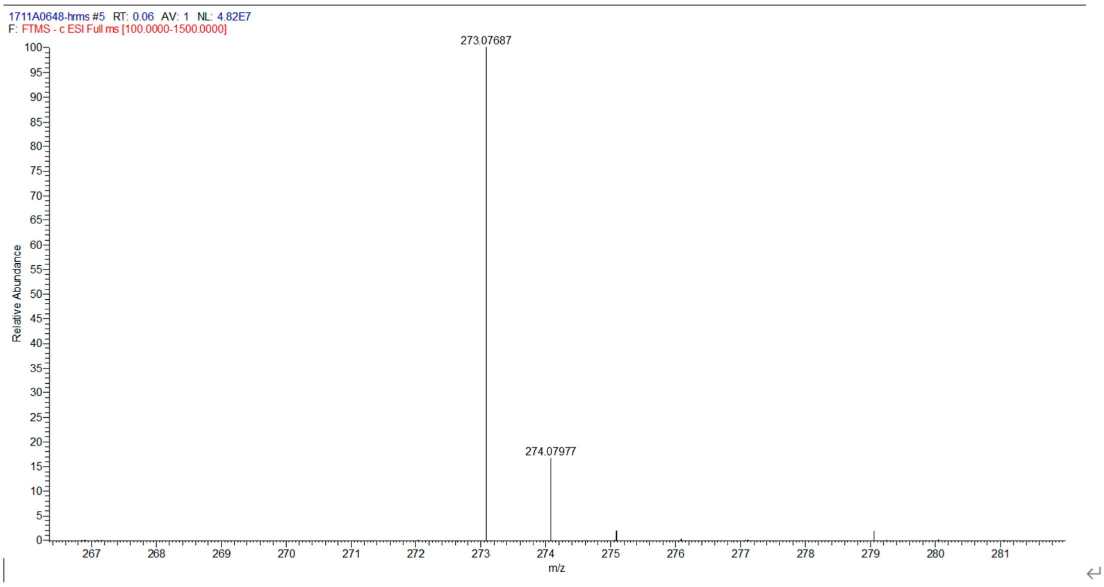

Figure S6. HRESIMS spectrum of compound **1**

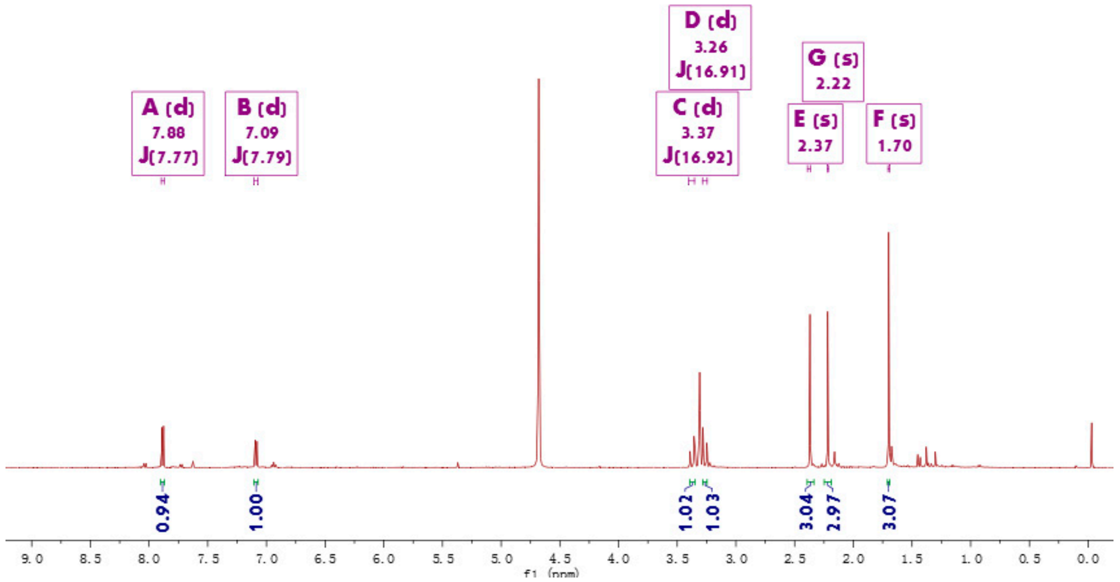

Figure S7. ^{1}H NMR spectrum of compound **2** (500 MHz, CD_3OD and $CDCl_3$).

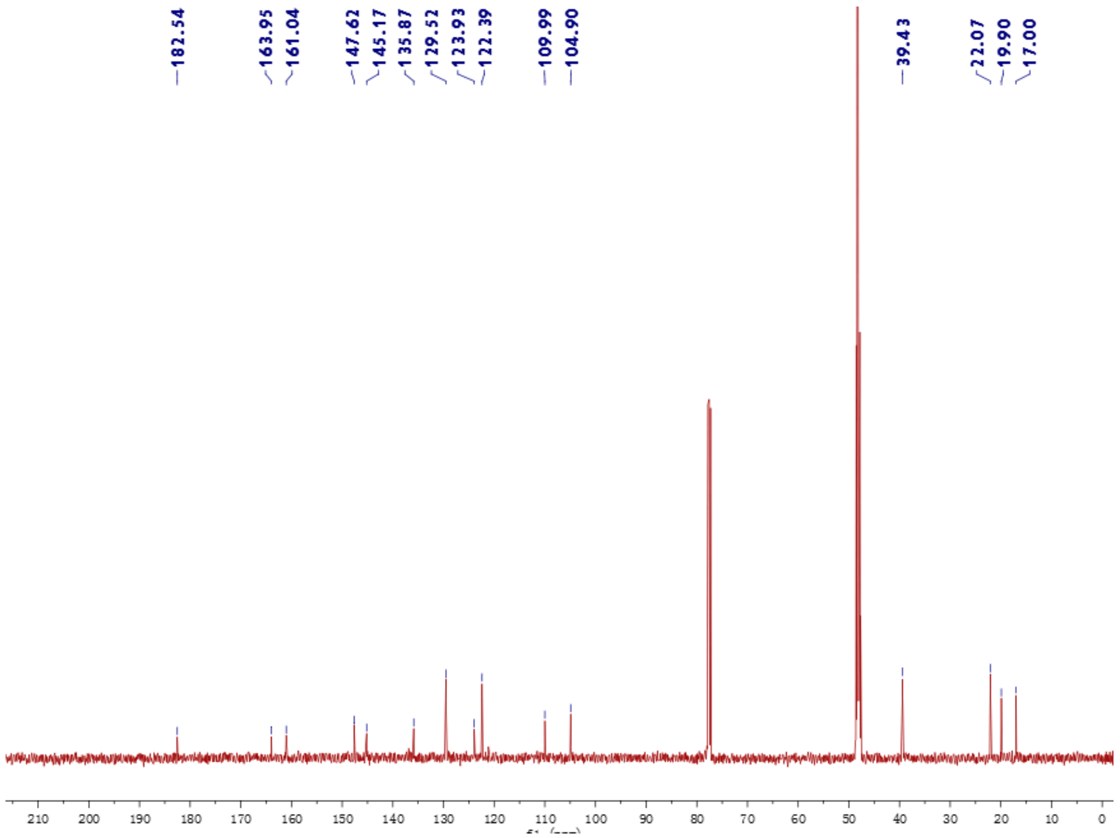

Figure S8. ^{13}C NMR spectrum of compound **2** (125 MHz, CD_3OD and $CDCl_3$).

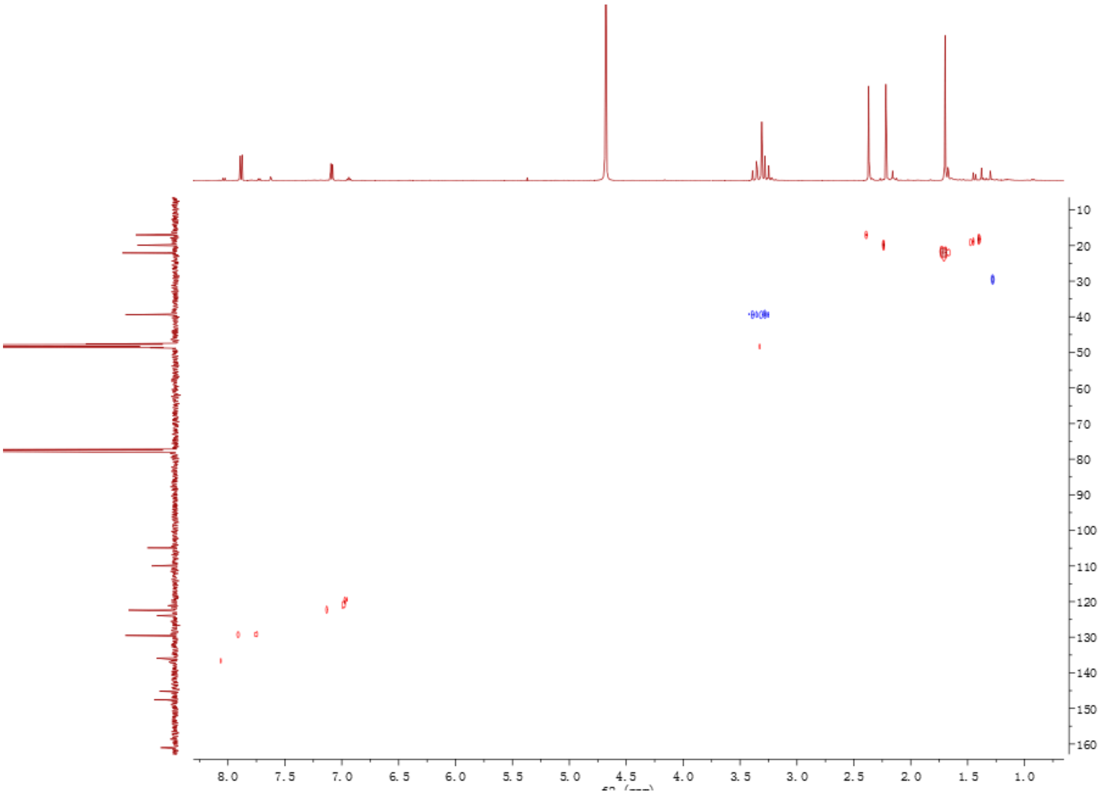

Figure S9. HSQC spectrum of compound **2**

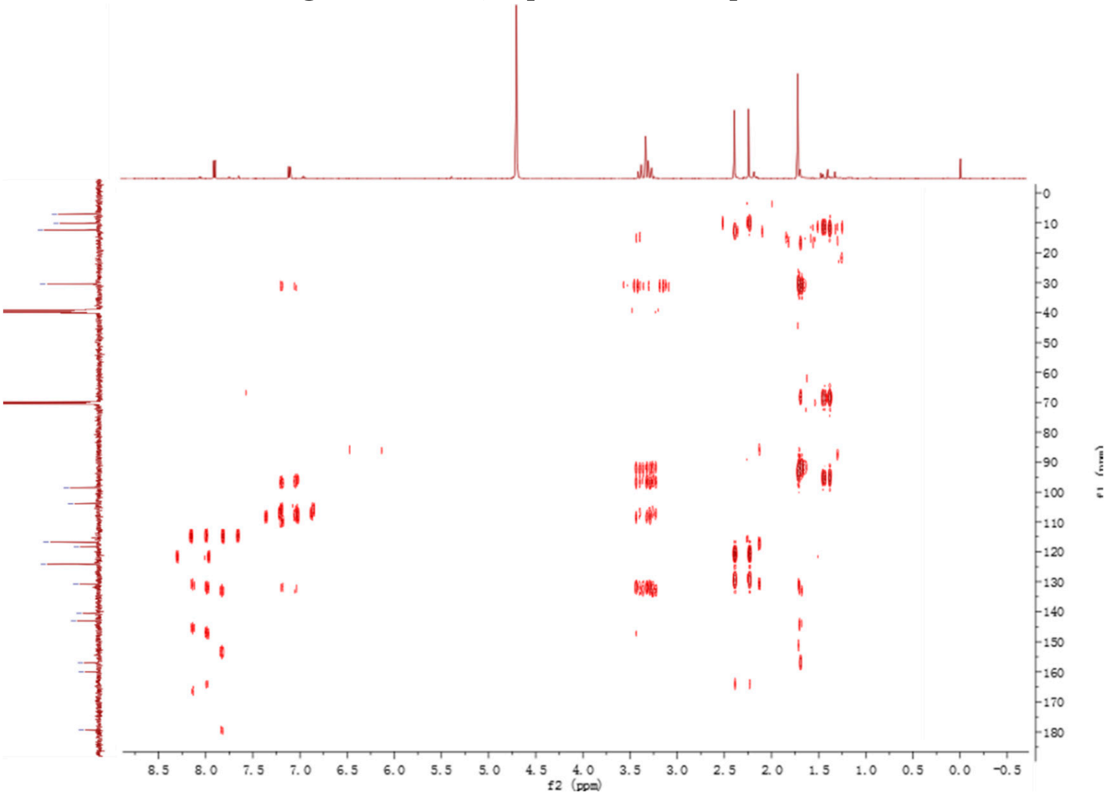

Figure S10. HMBC spectrum of compound **2**

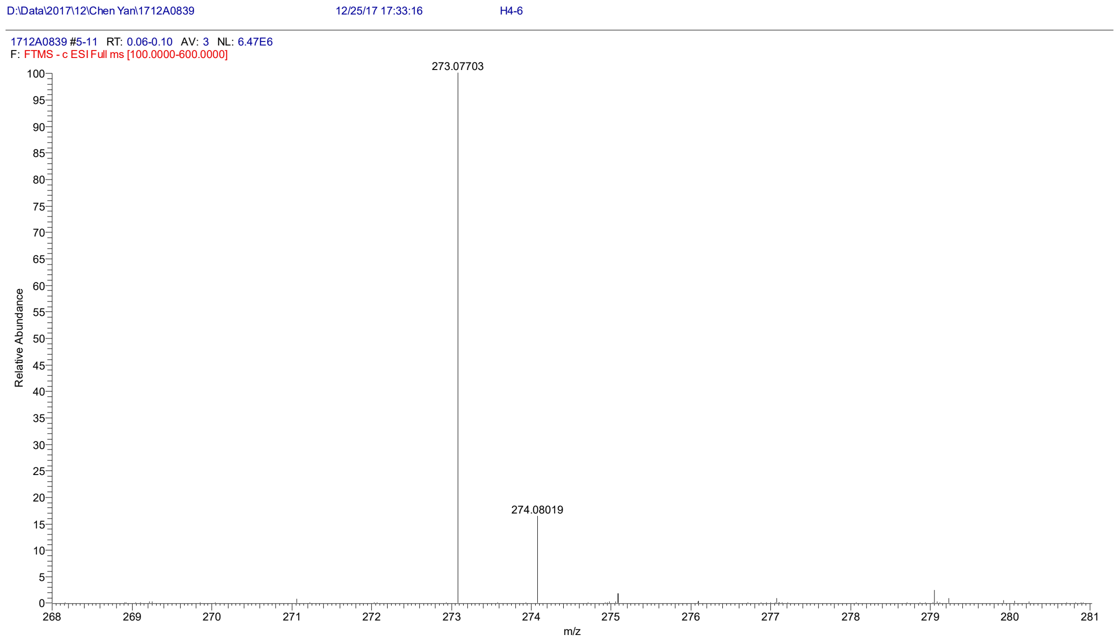

Figure S11. HRESIMS spectrum of compound **2**.

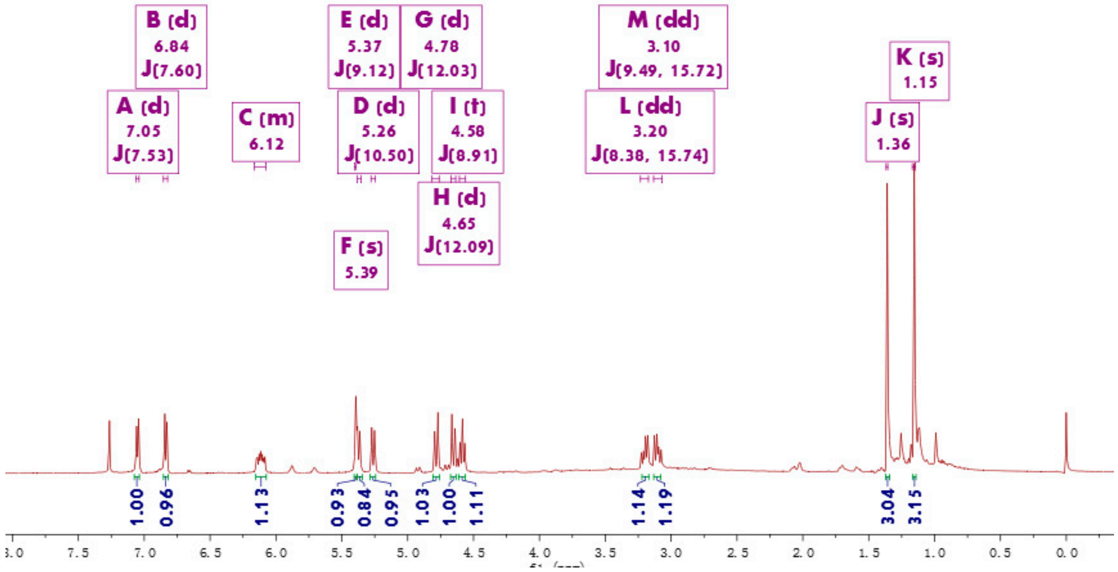

Figure S12. ^{1}H-NMR spectrum of compound **3** in CDCl3

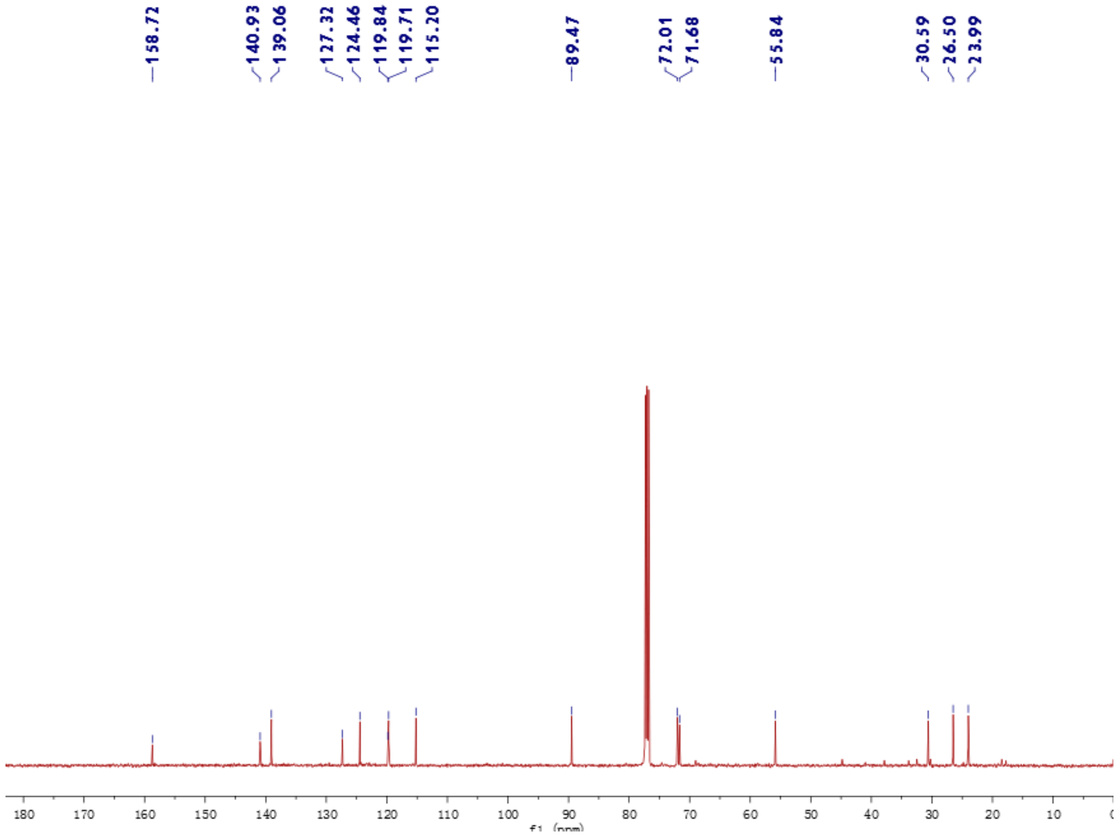

Figure S13. ^{13}C-NMR spectrum of compound **3** in $CDCl_3$

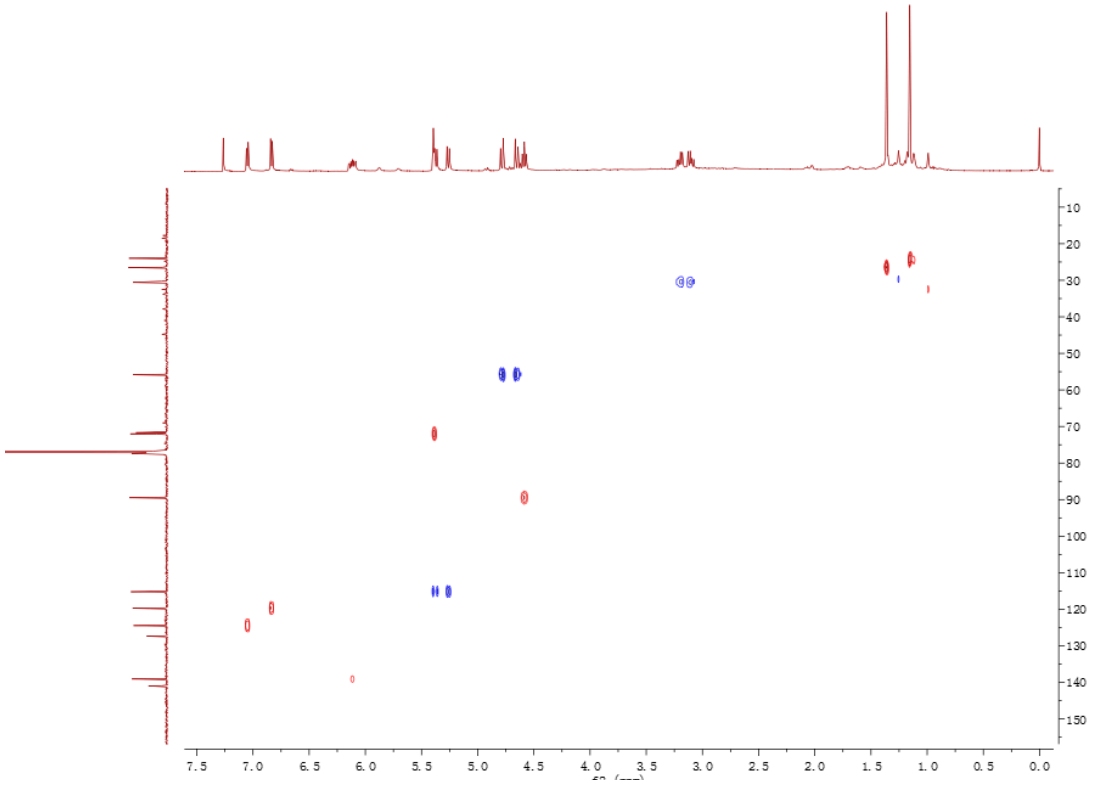

Figure S14 HSQC spectrum of compound **3**

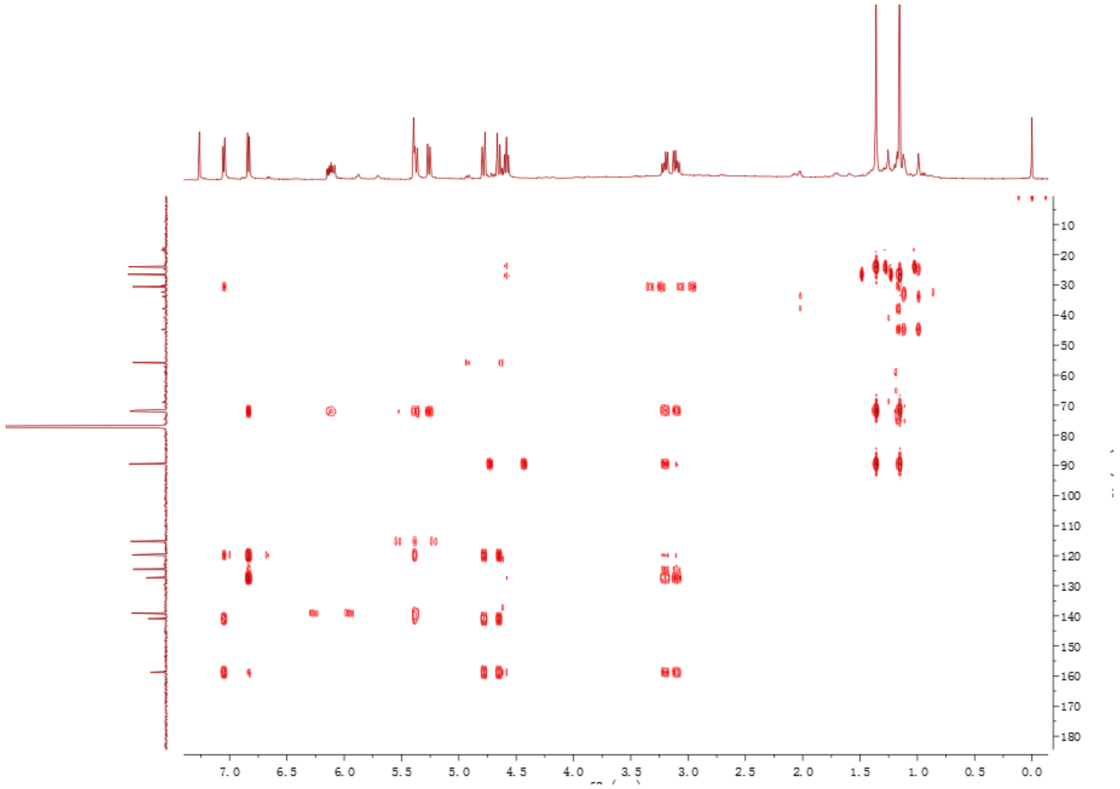

Figure S15. HMBC spectrum of compound **3**

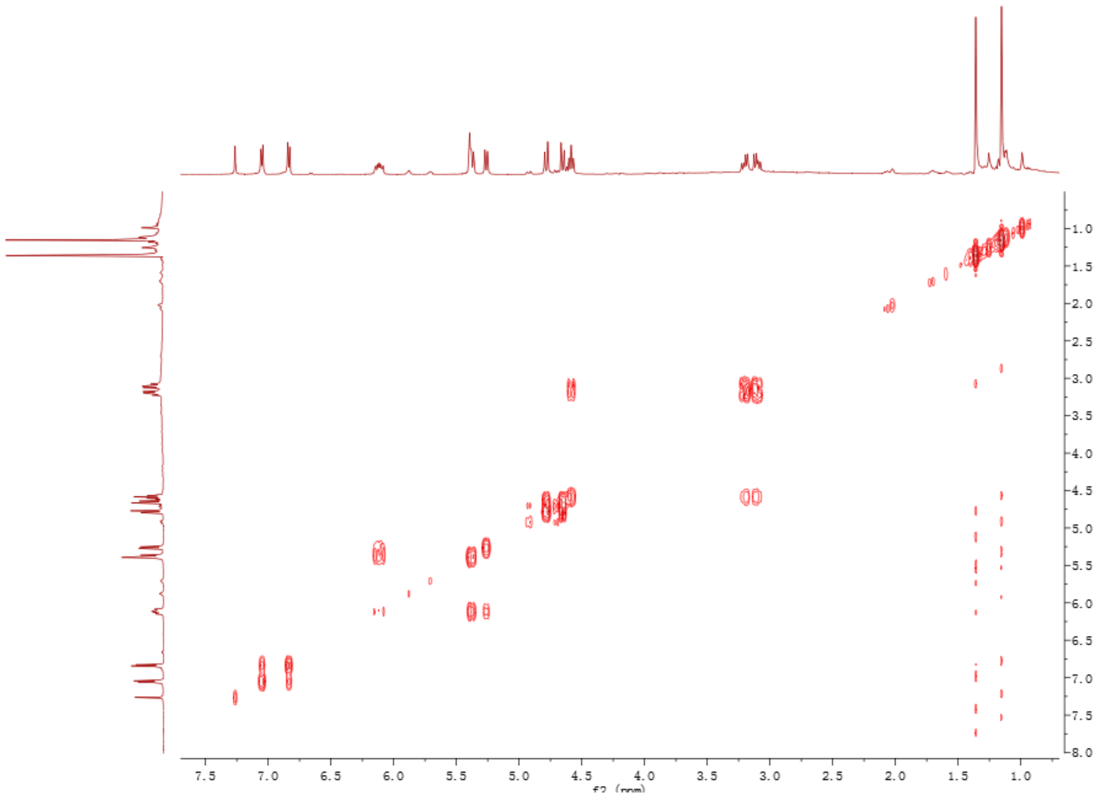

Figure S16. ^{1}H-^{1}H COSY spectrum of compound **3**

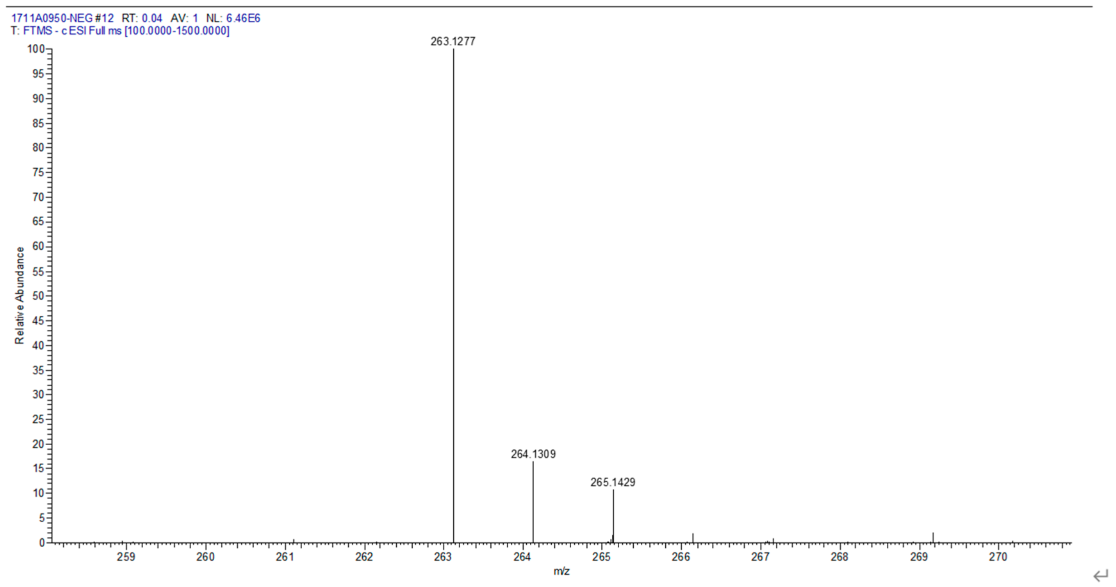

Figure S17. HRESIMS spectrum of compound **3**

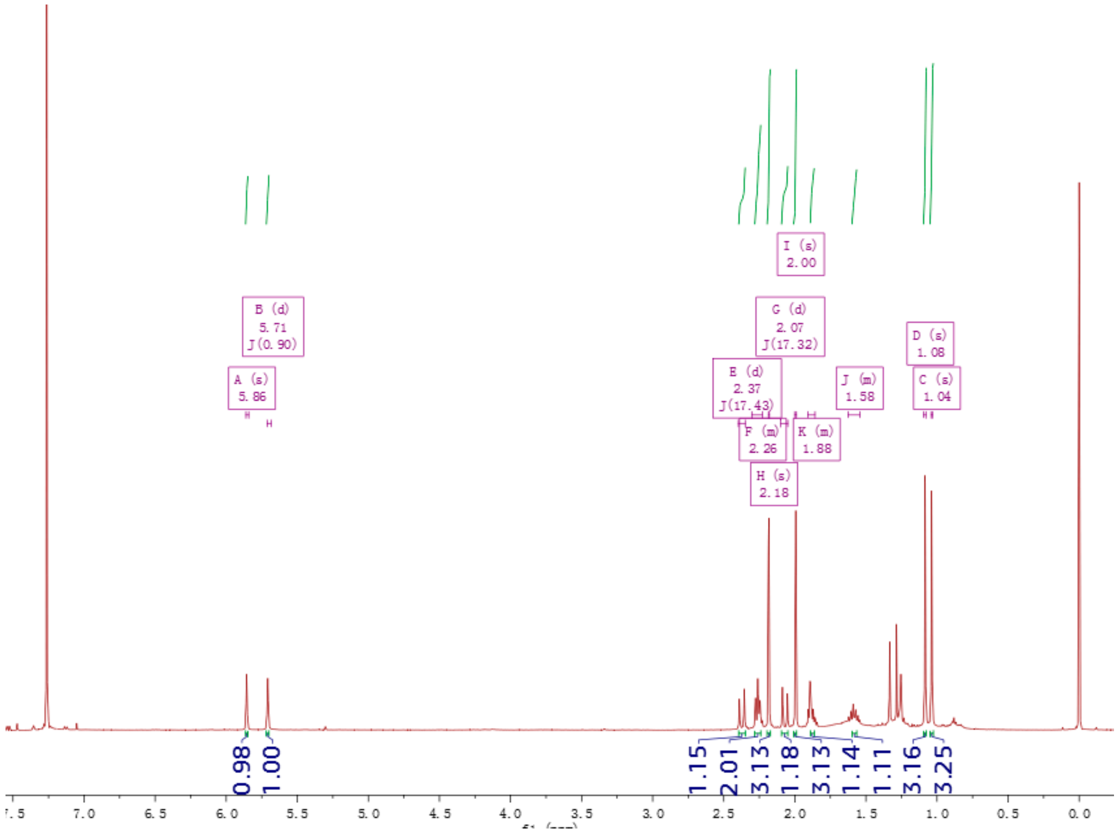

Figure S18 ^{1}H NMR spectrum of compound **4** in $CDCl_3$

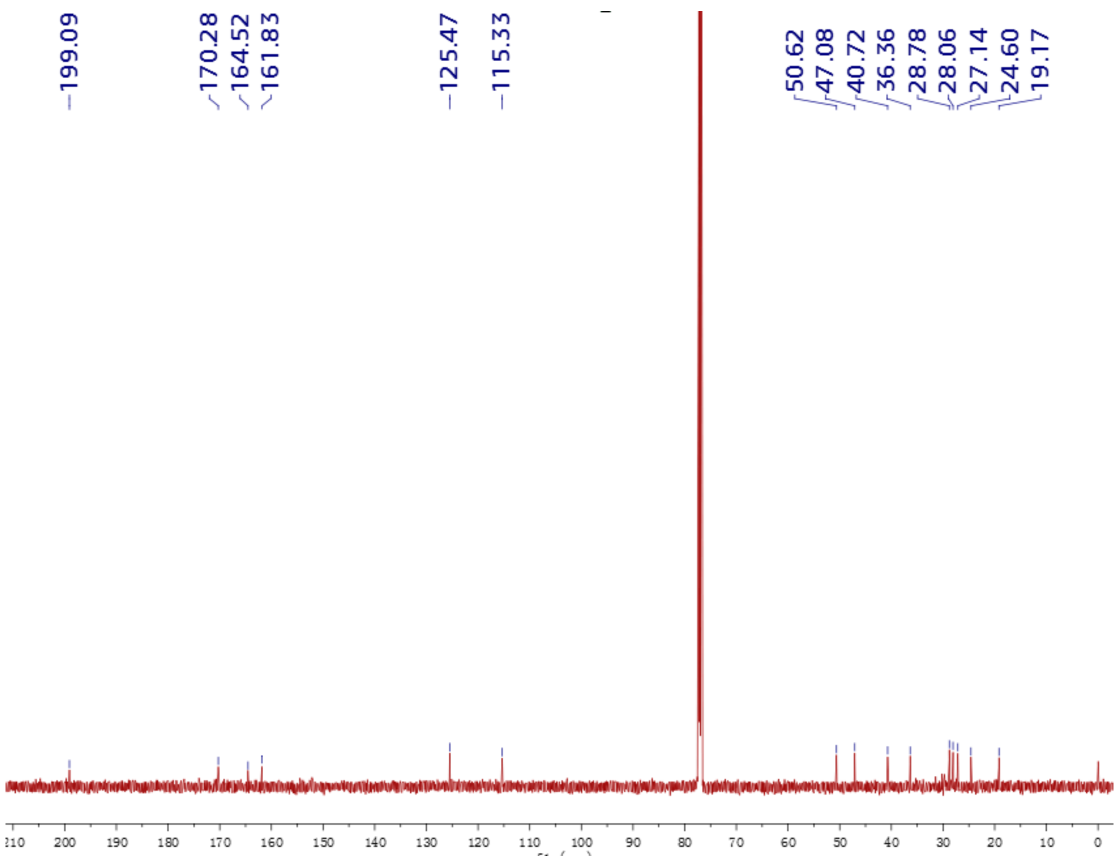

Figure S19. ^{13}C NMR spectrum of compound **4** in $CDCl_3$

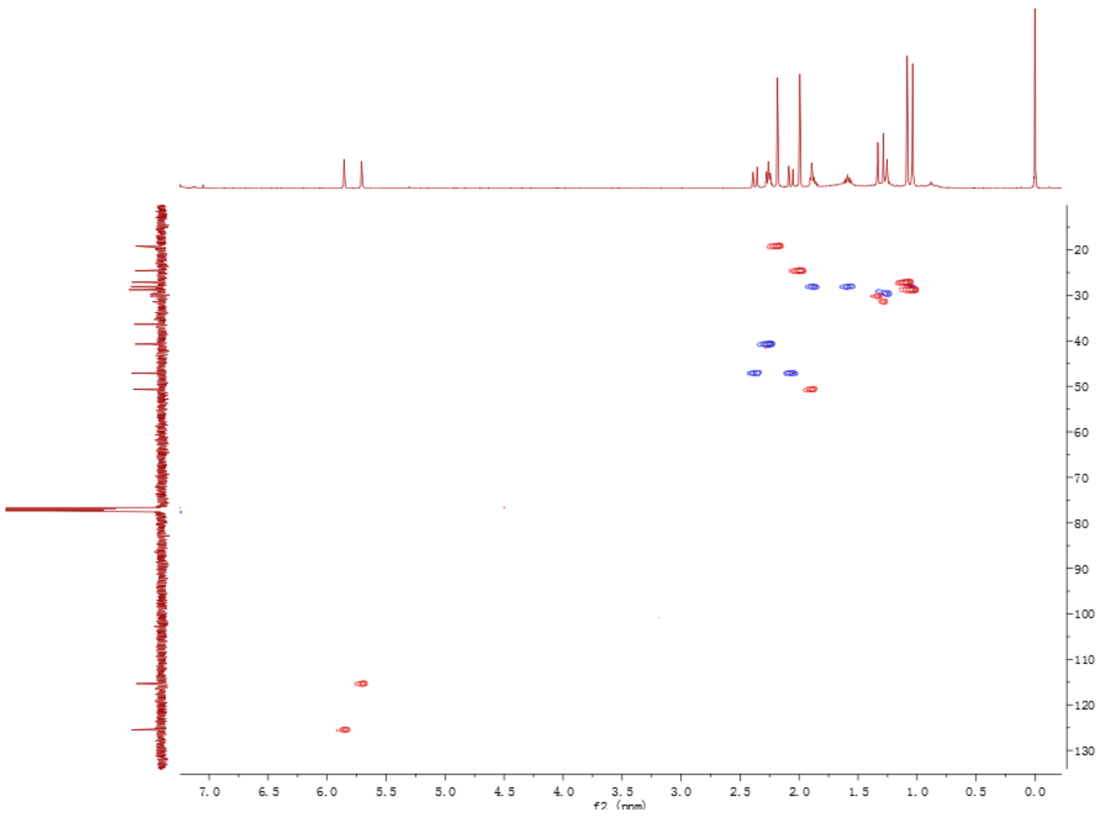

Figure S20. HSQC spectrum of compound **4**

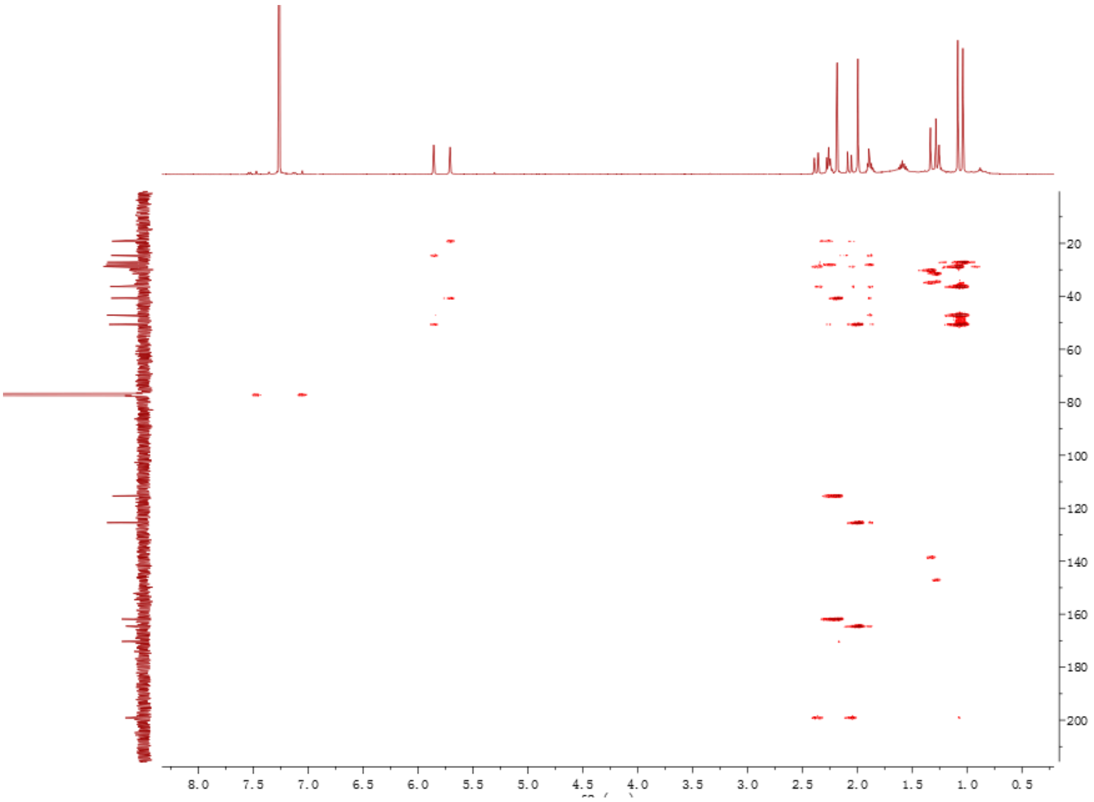

Figure S21. HMBC spectrum of compound **4**

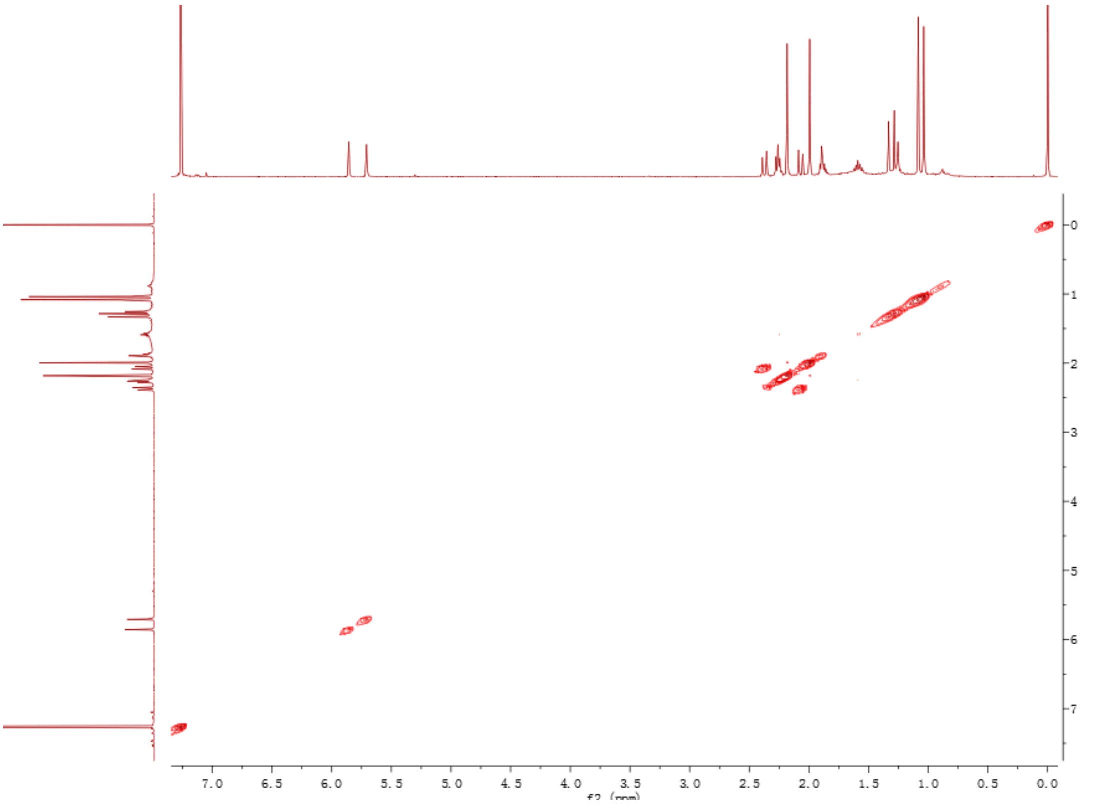

Figure S22. ^{1}H-^{1}H COSY spectrum of compound **4**

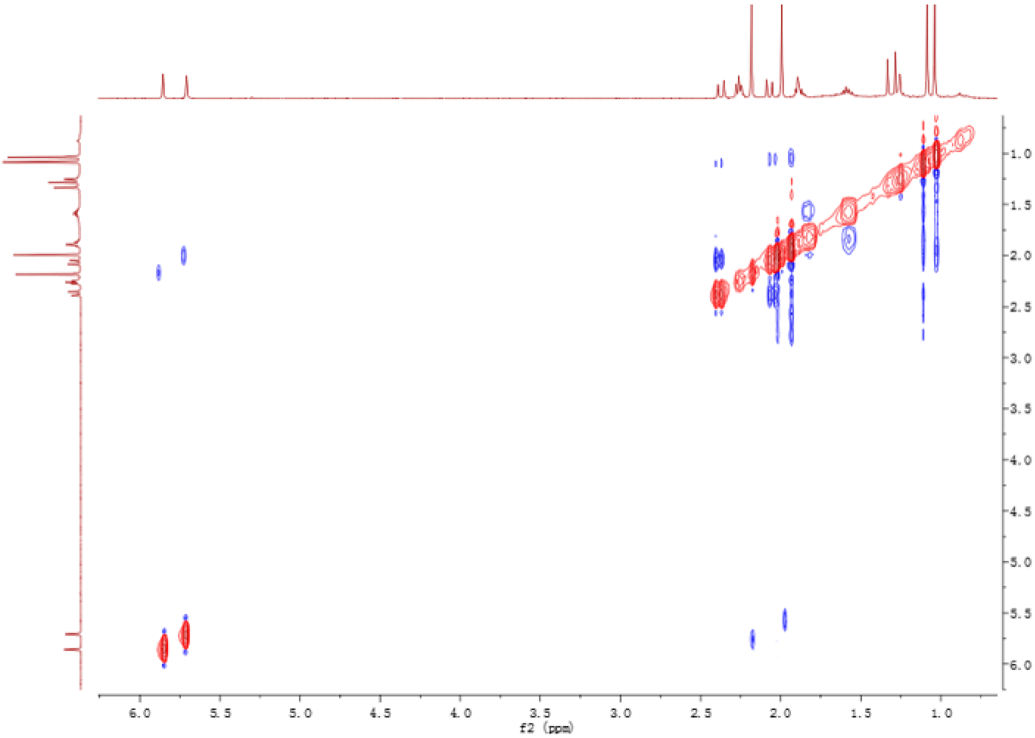

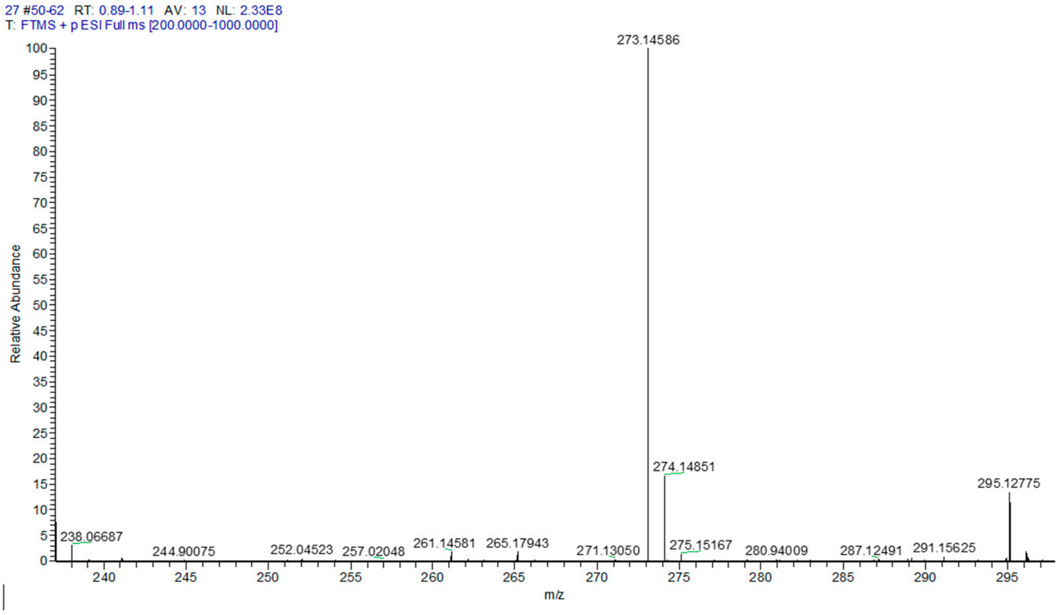

Figure S23. HRESIMS spectrum of compound **4**

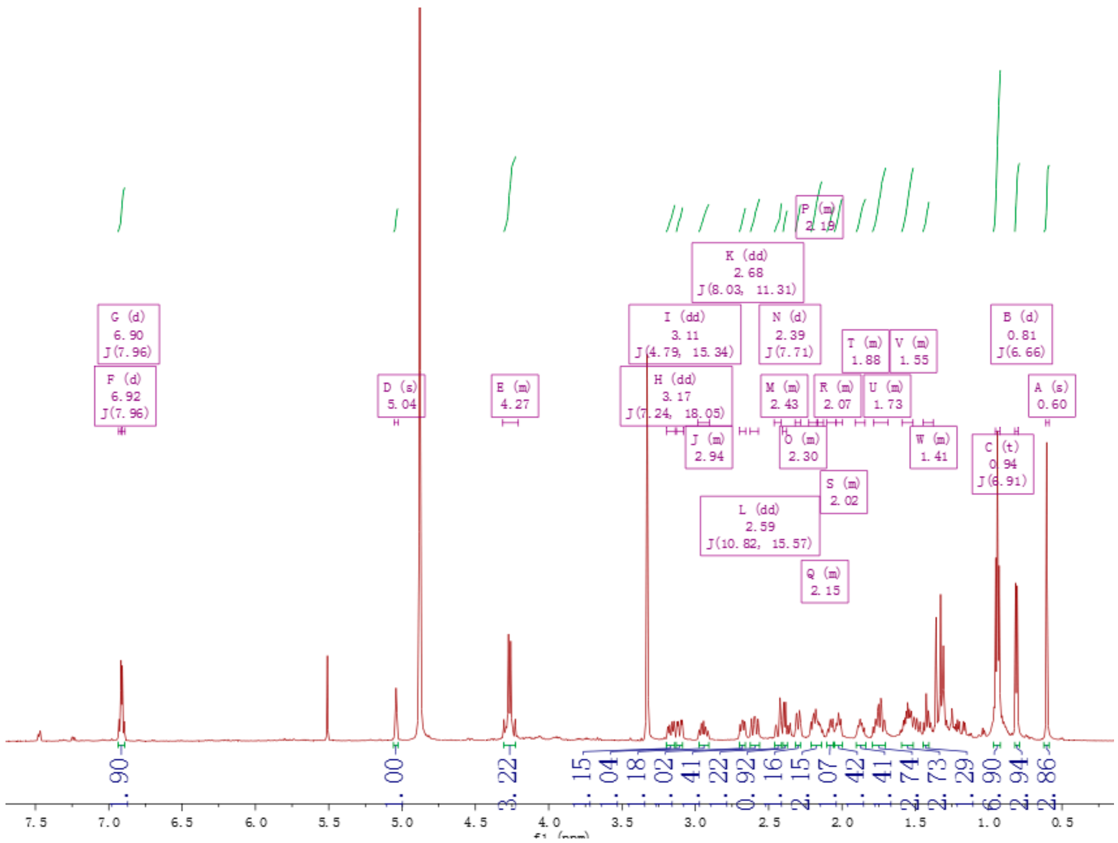

Figure S24. ^{1}H NMR spectrum of compound **5** (500 MHz, CD_3OD).

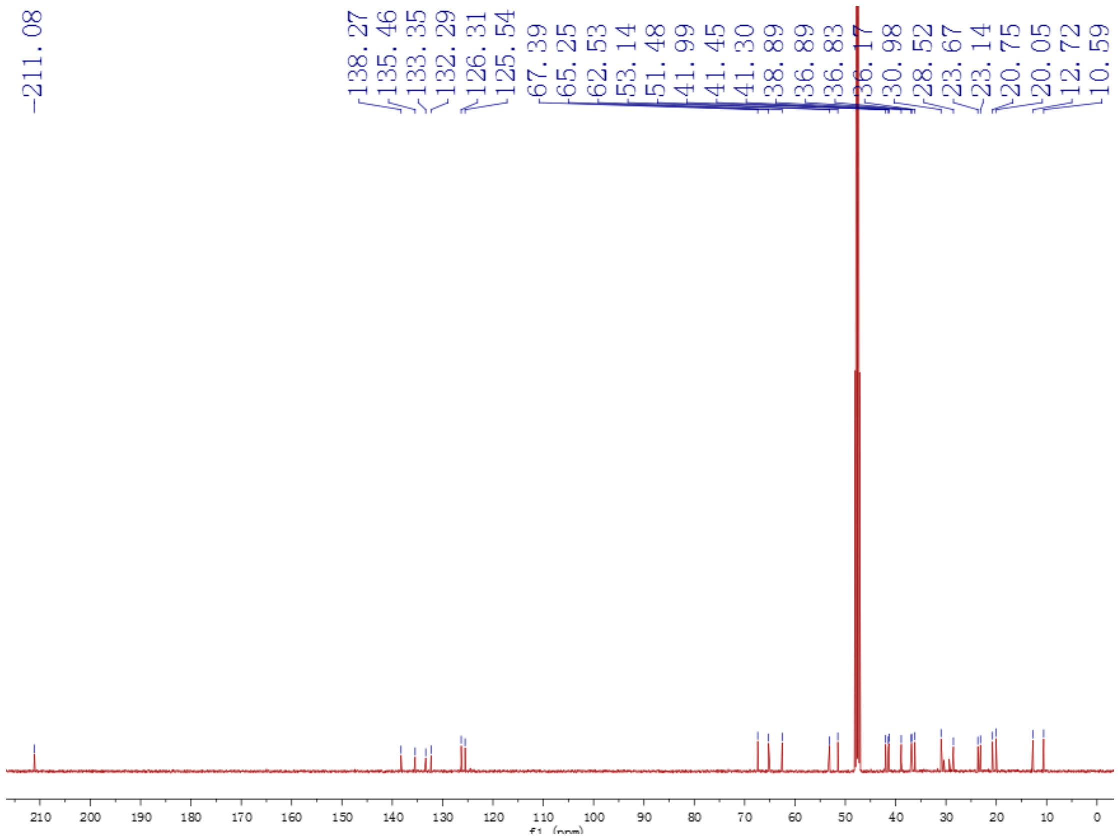

Figure S25. ^{13}C NMR spectrum of compound **5** (500 MHz, CD_3OD).

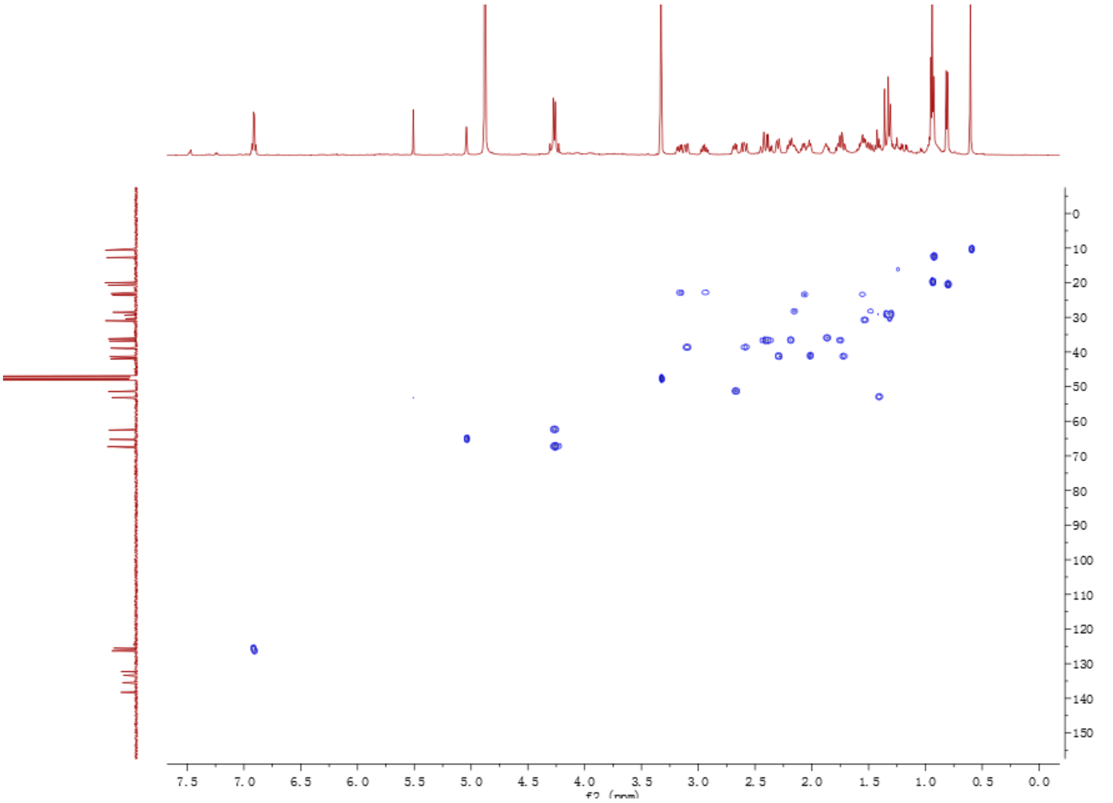

Figure S26. HSQC spectrum of compound **5**

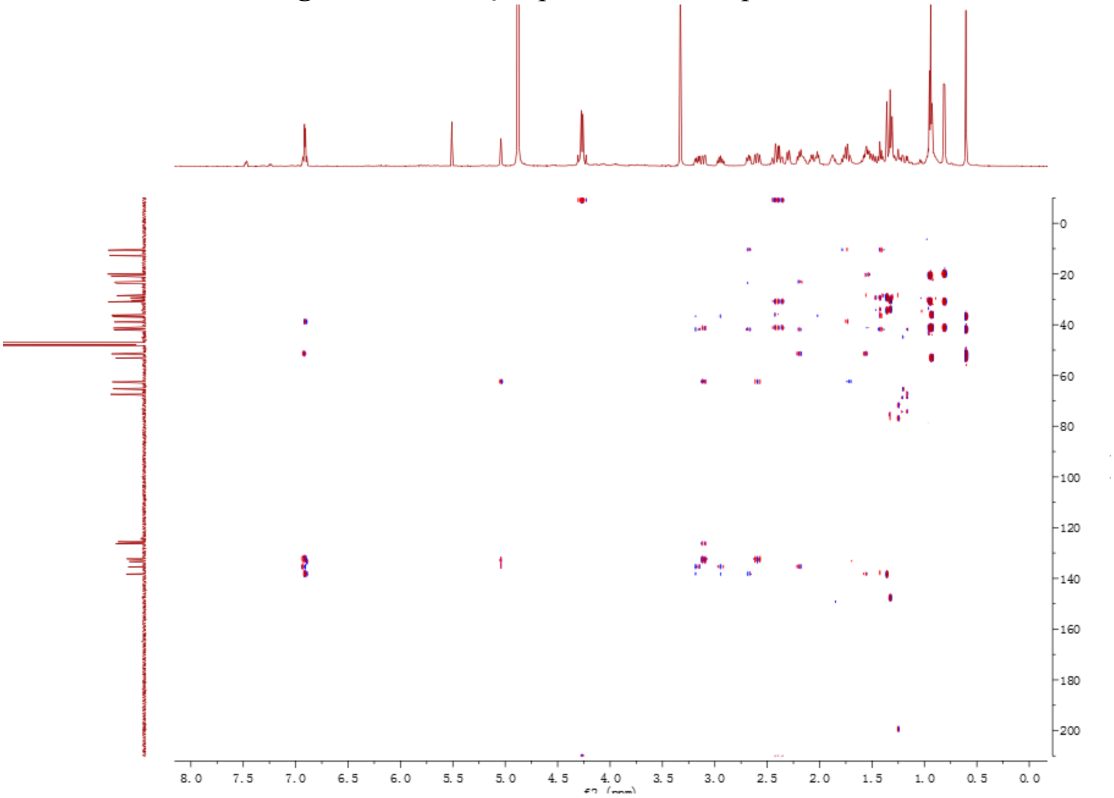

Figure S27. HMBC spectrum of compound **5**.

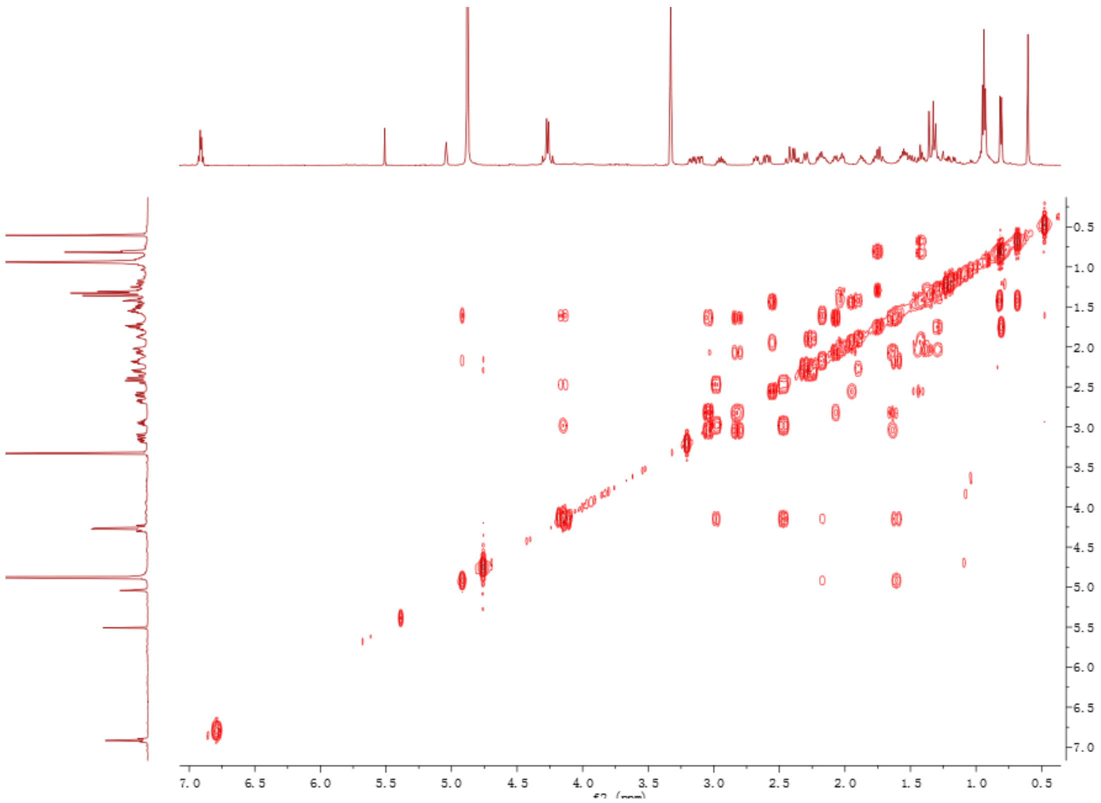

Figure S28. ^{1}H-^{1}H COSY spectrum of compound **5**

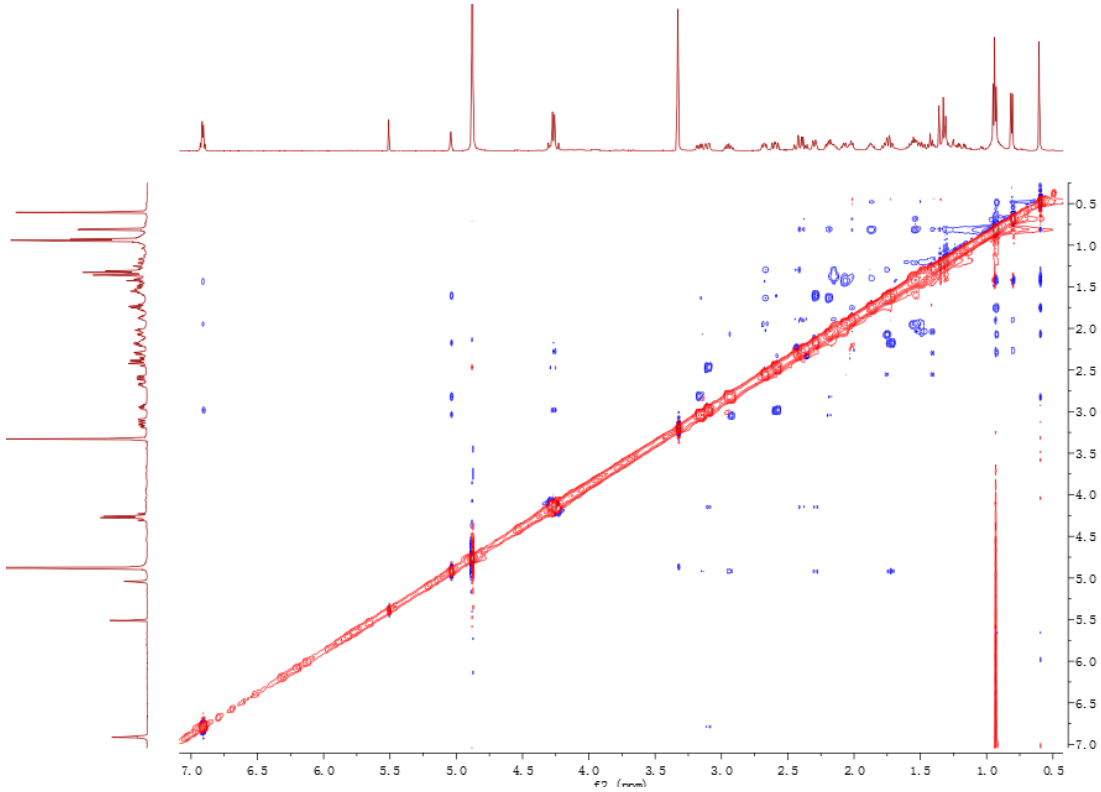

Figure S29. NOESY spectrum of compound **5**

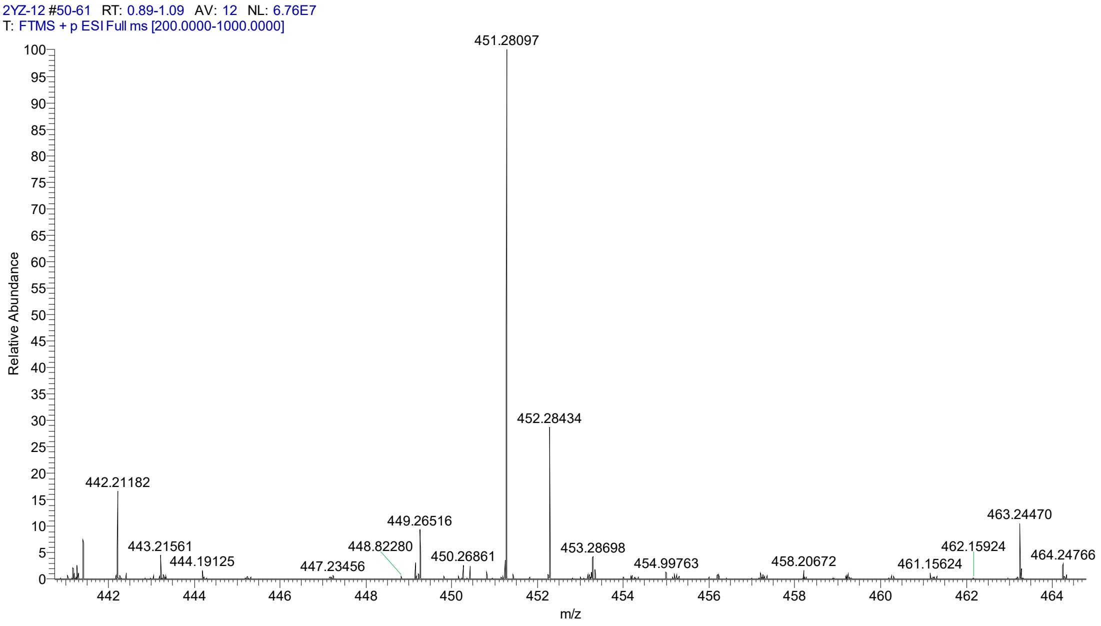

Figure S30. HRESIMS spectrum of compound **5**

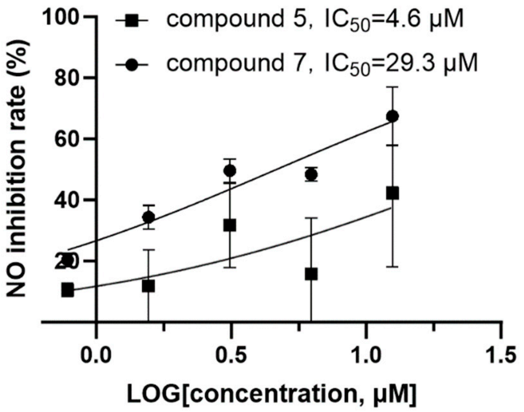

Figure S31. The IC_{50} value of compounds **5** and **7** against LPS-induced NO production in RAW264.7 cells were calculated and presented. Data were expressed as mean values ± SD, n = 3.

Table S1. The DP4+ evaluation of **3.**

Functional		Solvent?		Basis Set		Type of Data	
mPW1PW91		PCM		6-31G(d,p)		Unscaled Shifts	
		DP4+	0.00%	100.00%	-	-	-
Nuclei	sp2?	xperimenta	Isomer 1	Isomer 2	Isomer 3	Isomer 4	Isomer 5
C	x	127.2	129.3	128.8			
C	x	124.4	124.4	124.4			
C	x	119.6	121.0	120.8			
C	x	140.9	143.7	142.2			
C	x	119.8	120.2	120.5			
C	x	158.7	159.0	158.7			
C		89.4	90.6	90.5			
C		30.5	32.2	31.3			
C		71.6	72.4	72.5			
C		23.9	22.4	22.2			
C		26.4	26.6	26.5			
C		55.8	56.70	56.00			
C		72	76.00	73.30			
C	x	139	143.30	140.00			
C	x	115.2	113.00	113.70			
H	x	7.05	7.11	7.11			
H	x	6.83	6.77	6.76			
H		4.59	4.58	4.58			
H		3.1	3.01	3.02			
H		3.19	3.37	3.27			
H		1.15	1.19	1.20			
H		1.36	1.43	1.40			
H		4.65	4.82	4.68			
H		4.78	4.84	4.80			
H		5.39	5.28	5.42			
H		6.11	6.18	6.18			
H	x	5.25	5.41	5.3			
H	x	5.37	5.76	5.54			

Functional	Solvent?		Basis Set		Type of Data	
mPW1PW91	PCM		6-31G(d,p)		Unscaled Shifts	
	Isomer 1	Isomer 2	Isomer 3	Isomer 4	Isomer 5	Isomer 6
sDP4+ (H data)	0.11%	99.89%	-	-	-	-
sDP4+ (C data)	0.09%	99.91%	-	-	-	-
sDP4+ (all data)	0.00%	100.00%	-	-	-	-
uDP4+ (H data)	5.68%	94.32%	-	-	-	-
uDP4+ (C data)	4.65%	95.35%	-	-	-	-
uDP4+ (all data)	0.29%	99.71%	-	-	-	-
DP4+ (H data)	0.01%	99.99%	-	-	-	-
DP4+ (C data)	0.00%	100.00%	-	-	-	-
DP4+ (all data)	0.00%	100.00%	-	-	-	-
